# Photochemical Restoration of Light Sensitivity in the Degenerated Canine Retina

**DOI:** 10.3390/pharmaceutics14122711

**Published:** 2022-12-03

**Authors:** Sergei Nikonov, Natalia Dolgova, Raghavi Sudharsan, Ivan Tochitsky, Simone Iwabe, Jose-Manuel Guzman, Russell N. Van Gelder, Richard H. Kramer, Gustavo D. Aguirre, William A. Beltran

**Affiliations:** 1 Department of Neuroscience, Perelman School of Medicine, University of Pennsylvania, Philadelphia, PA 19104, USA; 2 Division of Experimental Retinal Therapies, Department of Clinical Sciences and Advanced Medicine, School of Veterinary Medicine, University of Pennsylvania, Philadelphia, PA 19104, USA; 3 Department of Molecular and Cell Biology, University of California, Berkeley, CA 90095, USA; 4 Department of Ophthalmology, Pathology, and Biological Structure, University of Washington, Seattle, WA 98195, USA

**Keywords:** photopharmacology, photoswitch, DENAQ, vision restoration, retinal degeneration, canine

## Abstract

Photopharmacological compounds such as azobenzene-based photoswitches have been shown to control the conductivity of ionic channels in a light-dependent manner and are considered a potential strategy to restore vision in patients with end-stage photoreceptor degeneration. Here, we report the effects of DENAQ, a second-generation azobenzene-based photoswitch on retinal ganglion cells (RGC) in canine retinas using multi-electrode array (MEA) recordings (from nine degenerated and six WT retinas). DENAQ treatment conferred increased light sensitivity to RGCs in degenerated canine retinas. RGC light responses were observed in degenerated retinas following ex vivo application of 1 mM DENAQ (*n* = 6) or after in vivo DENAQ injection (*n* = 3, 150 μL, 3–10 mM) using 455 nm light at intensities as low as 0.2 mW/cm^2^. The number of light-sensitive cells and the per cell response amplitude increased with light intensity up to the maximum tested intensity of 85 mW/cm^2^. Application of DENAQ to degenerated retinas with partially preserved cone function caused appearance of DENAQ-driven responses both in cone-driven and previously non-responsive RGCs, and disappearance of cone-driven responses. Repeated stimulation slowed activation and accelerated recovery of the DENAQ-driven responses. The latter is likely responsible for the delayed appearance of a response to 4 Hz flicker stimulation. Limited aqueous solubility of DENAQ results in focal drug aggregates associated with ocular toxicity. While this limits the therapeutic potential of DENAQ, more potent third-generation photoswitches may be more promising, especially when delivered in a slow-release formulation that prevents drug aggregation.

## 1. Introduction

Photoisomerization of the vitamin A derivative 11-*cis*-retinaldehyde to its *all-trans* conformation is the first biochemical reaction of the phototransduction cascade that takes place in retinal photoreceptor cells (rods and cones) and initiates the visual process [1]. Following photon catch, suppression of the dark current and interruption of the release of the neurotransmitter glutamate at the photoreceptor synaptic terminals stimulates second-order neurons. Through a complex process of visual processing within the inner retina, this visual information is subsequentially transmitted to retinal ganglion cells (RGCs) that encode and relay it in the form of action potentials to visual centers in the brain via the optic nerve.

Pathological conditions that progressively affect the function and/or structural integrity of photoreceptor cells (e.g., inherited retinal diseases (IRDs), age-related macular degeneration (AMD)) are common causes of irreversible blindness for which there is a dearth of therapeutic options. Several neuroprotective and corrective gene therapy strategies aimed at rescuing photoreceptors are currently under development or in clinical trials [2]. For patients who are at advanced stages of disease and for which rescuing photoreceptors is no longer an option, alternative approaches [3] include implantation of electronic retinal prosthesis [4], transplantation of stem cell-derived photoreceptors [5], delivery of optogenetic tools [6], and the use of optochemical compounds also called photoswitches [7].

The ability to alter the activity of membrane receptors and/or ion channels by using synthetic small molecule photoswitches whose conformational change can be controlled through light has given rise to the field of photopharmacology. Among the various classes of compounds, azobenzene-based photoswitches have been successfully used to control neuronal activity in the retina and restore light sensitivity and visual behavior in rodent models of IRDs [8,9,10,11]. While first-generation azobenzene photoswitches required exposure to two different wavelengths of light (one of them in the UV range) to transition from a cis–trans and trans–cis conformation, next-generation molecules can isomerize at a specific wavelength within the visual spectrum and thermally relax in the dark. DENAQ (diethylamine-azobenzene-quaternary ammonium) is one such second-generation channel blocker photoswitch that exhibits trans to cis photoisomerization with visible light (450–550 nm) and relaxes rapidly in the dark to the trans configuration that blocks cation channels [12]. Intravitreal injection of DENAQ in strains of mutant blind mice enables light-dependent firing of action potential in RGCs and restoration of visually guided behavior [9]. DENAQ confers photosensitivity to rodent RGCs in a degeneration-dependent manner by entering the cell through P2X receptors and acting on HCN channels [13].

In an attempt to assess the translational potential of this photoswitch for vision restoration in patients with end-stage photoreceptor degeneration, this study was conducted to evaluate whether DENAQ confers sensitivity to canine RGCs, determine its efficacy when delivered intravitreally in a large animal eye, and assess its ocular tolerance.

## 2. Material and Methods

### 2.1. Animals

A total number of 30 dogs were used for these studies (Supplementary Tables S1 and S2). These included 18 WT and 12 mutant dogs at advanced stages of retinal degeneration caused by mutations in either *PDE6B* (rcd1 and crd1 dogs) or *RPGR* (XLPRA2 dogs) [14,15,16]. All three mutant models undergo an early onset and rapidly progressive loss of photoreceptors but retain inner retinal neurons even in late-stage disease [15,17,18]. With the exception of 3 WT beagles purchased from a commercial vendor, the dogs were bred and maintained at the University of Pennsylvania Retinal Disease Studies Facility. Studies were carried out in strict accordance with the recommendations in the Guide for the Care and Use of Laboratory Animals of the National Institutes of Health in compliance with the USDA’s Animal Welfare Act, Animal Welfare Regulations, and the ARVO Statement for the Use of Animals in Ophthalmic and Vision Research. The protocols were approved by the Institutional Animal Care and Use Committee of the University of Pennsylvania.

### 2.2. Intravitreal Injections with and without Prior Vitrectomy

All surgical procedures were carried out under sterile conditions in a dedicated veterinary ophthalmic surgical suite. Dogs were premedicated for anesthesia with atropine sulfate (0.02–0.04 mg/kg SQ), acepromazine maleate (0.5 mg/kg SQ), and were anesthetized with a mixture of inhaled isoflurane with oxygen delivered through a semi-closed system. The eyelids and conjunctival fornices were prepped with povidone-iodine 1% solution. A lidocaine eyelid block followed by a retrobulbar injection of saline solution, and the placement of stay sutures enabled proper positioning of the globe. An operating microscope (Zeiss OPMI 6, Carl Zeiss Inc, Oberkochen, Germany) equipped with a digital camera (Sony Alpha NEX-5N) was used for the surgery. Under direct visualization of the posterior segment through a magnifying vitrectomy contact lens (Acrivet Vit.Lens; Acrivet, Salt Lake City, UT, USA), the needle of 30-gauge insulin syringe was introduced into the mid-vitreous to inject 150 μL of a DENAQ (concentration range: 0.1–10 mM) or vehicle solution. An anterior chamber paracentesis was immediately performed after the injection to prevent any increase in intraocular pressure. In a subset of dogs, a standard three-port pars plana posterior vitrectomy was performed, using a vitreoretinal unit (Stellaris PC, Bausch & Lomb, Bridgewater, NJ, USA) and 23- or 25-gauge ports and instruments, 2 to 63 weeks prior to the injection of 150 μL of a DENAQ (concentration range: 1–10 mM) or vehicle solution into the vitreal cavity. Ophthalmic examinations including biomicroscopy, tonometry, and indirect ophthalmoscopy were conducted on a regular basis throughout the injection termination time interval. In a subset of animals, fundus photography (RetCam shuttle, Natus, Plesanton, CA, USA), ocular ultrasonography (SonoScape S2, Nanshan, Shenzen, China), and/or non-invasive retinal imaging by confocal scanning laser ophthalmoscopy and spectral domain optical coherence tomography (Spectralis HRA + OCT, Heidelberg, Germany) was performed. At termination, ocular tissues were collected after euthanasia with intravenous injection of euthanasia solution (Euthasol; Virbac, Ft. Worth, TX, USA). During the study, all efforts were made to improve animal welfare and minimize discomfort. 

### 2.3. MEA Recording

MEA recordings were performed according to previously published methods [19] with some modifications. Briefly, 5 mm diameter punches of neuroretina were excised from the *area centralis* under room light and attached to a polyester membrane (using Corning 3450 cell culture transwells) on the photoreceptor side with gentle suction. The membrane with the attached retina was placed in the recording chamber ganglion cell-side down with the membrane covering it from above. Finally, a harp (0.8 g, HSG-MEA-5BD, ALA Scientific, Farmingdale, NY, USA) was placed on top of the membrane to improve retinal contact with the electrodes. Custom Ringer’s solution (119 mM NaCl, 2.5 mM KCl, 1 mM KH_2_PO_4_, 1.3 mM MgCl_2_, 2.5 mM CaCl_2_, 26.2 mM NaHCO_3_, 20 mM d-glucose in ddH_2_O) saturated with 95%O_2_/5%CO_2_) was used for dissection and to perfuse the recording chamber, the temperature of the perfusate was increased to 37 °C before starting the recording. Standard MEA arrays (60MEA200/30IR–ITO–G) were used in the earlier and perforated arrays (pMEA200/30iR-Ti) in the latter experiments. Both were 60 electrode arrays with 30 μm electrode diameter and 200 μm inter-electrode distance (Multichannel Systems, Reutlingen, Germany).

A MEA1060-Inv MEA amplifier (Multichannel Systems, Reutlingen, Germany) mounted on the inverted microscope (Olympus IX51, Olympus Corporation of America, Center Valley, PA, USA) was used to record ganglion cell activity. Data digitized at 10 KHz were collected using custom made acquisition software developed in LabView (National Instruments, Austin, TX, USA). For each channel all recorded data were consecutively combined in a single dataset using custom code developed in Matlab (MathWorks, Natick, MA, USA) and Plexon Offline Sorter (Plexon, Inc., Dallas, TX, USA) was used for spike sorting on the resulting dataset. Custom Matlab based code was used for further data analysis. Low firing cells (producing less than 30 spikes for the 150 s duration of the stimulation series, see below) were excluded from the analysis.

For ex vivo DENAQ application retinas were incubated (without perfusion) with Ringer’s solution containing 1 mM of DENAQ for 20 min. For retinas treated by in vivo intravitreal injection, DENAQ was administered at concentration of either 3 mM (*retinas 13 and 14*), or 10 mM (*retina 15*) 48 h prior to the MEA recording session. To block synaptic transmission from second order neurons to RGCs, retinas were perfused with Ringer’s containing a cocktail of synaptic blockers (10 μM AP4, 40 μM DNQX, 30 μM AP5, 10 μM SR-95531 (GABAzine), 50 μM TPMPA, 10 μM strychnine, 50 μM tubocurarine) as previously used in canine retinas [19].

Light stimulation system included a custom LED-based light source (455 nm 75 lm Diamond Dragon series LED, Osram Opto Semiconductors/Digi-Key, Thief River Falls, MN) with TechSpec coated plano-convex Lens 25 mm Dia., 25 mm FL, 455 nm CWL, 10 nm FWHM interference filter (Edmund Optics, Barrington, NJ, USA), and LED driving circuit under control of Clampex software and Digidata 1440 digitizer (Molecular Devices, San Jose, CA, USA). Calibrated full-field flashes of 455 nm light were delivered to the retina from below the stage through the optical port of the microscope and a 4× microscope objective. Duration of each stimulation series was 150 s of which 100 s were used for light stimulation (3 s before first flash onset). Unless otherwise noted, stimulation series included 1 s or 5 s flashes delivered at 0.1 Hz (10 flashes per stimulation series) or 50 ms flashes delivered at 4 Hz (flicker stimulation, 400 flashes per series). Light intensities were regularly measured between experiments at the retinal plane using a calibrated photodiode (OSI Optoelectronics, Hawthorne, CA, USA). The timing accuracy of flash delivery was better than 0.1 ms.

### 2.4. qRT-PCR

Archival cDNA preparations that originated from neuroretina of 3 WT dogs (females, age: 24 weeks), 3 *PDE6B*/rcd1 affected (age: 22 weeks), and 3 *RPGR*/xlpra2 affected dogs (females; age: 41 weeks) dogs were used (Supplementary Table S3). Because of different degeneration rates, the *PDE6B*/rcd1 and *RPGR*/xlpra2 dogs have comparable disease stages at the time periods studied [20]. Details on sample preparation have been published previously [20]. Gene-specific primer pairs (Supplementary Table S4) for qRT-PCR were designed using PrimerQuest Tool and ordered from Integrated DNA technologies. qRT-PCR was performed on a ViiA 7 Real-Time PCR System (Applied Biosystems, Waltham, MA, USA) using 5 ng of cDNA, 250 nM of each primer, and SYBR Green PCR mastermix (Life Technologies, Carlsbad, CA, USA). Each sample was tested in triplicates. Relative comparison of gene expression was performed using comparative delta Ct method. GAPDH was used as an endogenous control for normalization. Fold change was calculated as 2^−(ΔΔCt)^. Statistical significance was calculated by an unpaired heteroscedastic *t*-test using a one-tailed distribution.

### 2.5. Histology and Immunohistochemistry

At the end of the MEA recordings, ocular tissues (including the neuroretina punches placed on the MEA array, as well as the remaining posterior ocular cups) were fixed overnight in 4% paraformaldehyde, cryoprotected by incubation in 15% and 30% sucrose in balanced salt solution and embedded in optimal cutting media. Ten-micron-thick cryosections were H&E stained or used for fluorescent immunohistochemistry to label cones (goat anti-human cone arrestin, Beltran Lab, 1:100), and RGCs (mouse anti-Brn3a, Chemicon cat #MAB1585, 1:100) as previously described [21]. A subset of the retinal punches used on the MEA array were prepared instead as flatmounts to perform Brn3a fluorescent immunohistochemistry. Following termination of the dogs that were used to assess the ocular tolerance of intravitreally-injected DENAQ (or vehicle), their eyes were fixed for 48 h in a solution of alcoholic Bouin’s fixative and paraffin embedded. Five to six micron thick sagittal sections were H&E stained. Sections were examined under confocal (Leica TCS SP5; Leica Microsystems, Buffalo Grove, IL, USA) or wide field microscopic systems (Axioplan; Carl Zeiss Meditec, Dublin, CA, USA).

## 3. Results

### 3.1. MEA Analysis following Ex Vivo Application of DENAQ on Degenerated and WT Retinal Explants

**Figure 1:**
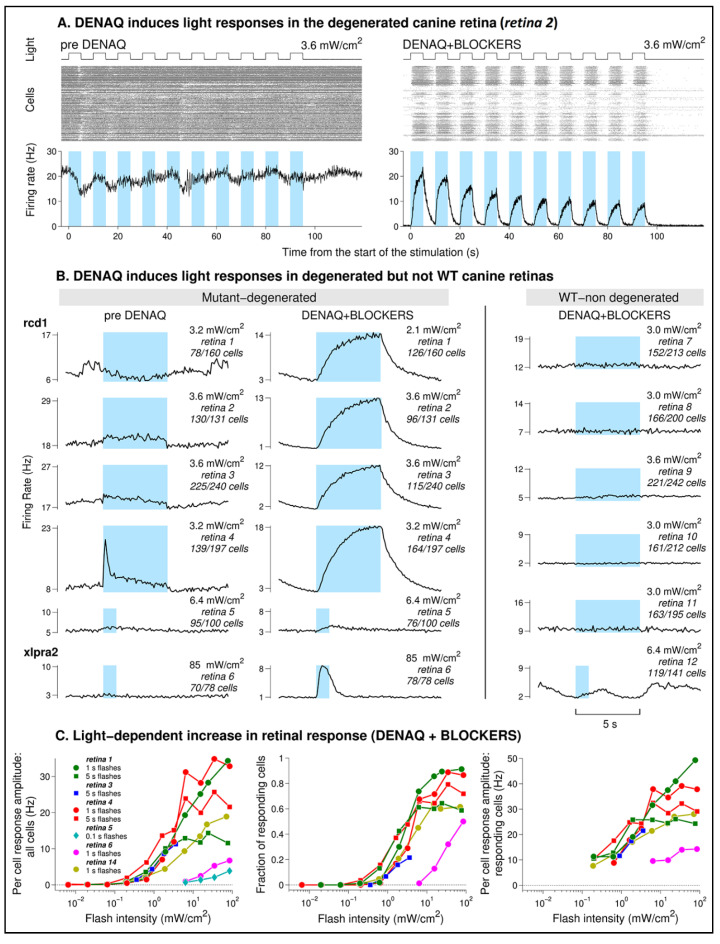
DENAQ induces strong light-dependent firing in the degenerated but not in the WT canine retina. (**A**) Raster plots and averaged (per cell) firing rates obtained during perfusion with Ringer’s solution before DENAQ application and with Ringer’s solution containing synaptic blockers following 20 min of exposure to 1 mM DENAQ (*retina 2*). The same stimulation series was used under both conditions, upper traces (“Light”) show the time course of light stimulation. On the raster plots (“Cells”) each dot corresponds to an action potential firing event, and each line represents firing from an individual retinal ganglion cell. Per cell averaged firing rates were calculated using 100 ms time bin, colored blue bars indicate light flashes. Cells producing less than 30 spikes in 150 s (total stimulation series duration) were excluded from the analysis. (**B**) Per cell and per flash averaged responses from 6 degenerated and 6 WT retinas. Left and middle columns show responses from degenerated retinas before and after ex vivo DENAQ application under similar light stimulation conditions. Right column shows responses of WT retinas recorded after ex vivo DENAQ application. Colored blue bars indicate light stimulation. Number of cells per retina is reported as number of cells which responses were averaged to calculate retinal response over total number of cells identified in the retina (responses of cells producing less than 30 spikes per series were excluded from the averaging). (**C**) Light intensity dependence of the parameters of the DENAQ-driven responses. Left plot: amplitudes of the per cell per flash averaged responses (like those illustrated on panel B) counting all cells producing more than 30 spikes per stimulation series. Response amplitudes were measured at the time of flash termination from degenerated retinas perfused with synaptic blockers following ex vivo DENAQ application (*retinas 1*, *3*, *4*, *5*, *6*) or after in vivo DENAQ injection (*retina 14*). Middle plot: fraction of light sensitive cells for each retina (defined as the number of cells with responses at or above 7.5 Hz divided by the total cell count for each retina, *retina 5* was excluded from the analysis due to noisy single cell responses). Right plot: per cell per flash averaged response amplitudes of responding cells.

Ex vivo application of DENAQ (1 mM of DENAQ for 20 min, see methods) on retinal explants resulted in the appearance of robust light-dependent firing of RGCs in the degenerated retinas (Figure 1A). Light responses were absent before DENAQ application and were observed after DENAQ application during continuous perfusion with Ringer’s solution containing synaptic blockers, thus confirming the existence of a direct DENAQ-driven light-dependent activation of RGCs. 

Averaged light responses observed in six degenerated retinas before DENAQ application (“pre DENAQ”) and during perfusion with synaptic blockers following DENAQ application (“DENAQ + BLOCKERS”) are illustrated in Figure 1B. All retinas except one (*retina 4*) did not show any significant light dependent firing before DENAQ application. DENAQ induced strong light responses in four rcd1 retinas at light intensities in the range of 2.1–3.6 mW/cm^2^. A much smaller response was observed from the older (7 y of age) rcd1 retina (*retina 5*) with a ~2× brighter light suggesting that older/more degenerated retinas might be less sensitive to DENAQ. The single xlpra2 retina (*retina 6*), used at an age at which the stage of disease is less advanced than in three of the rcd1 retinas (*retinas 2–4*), exhibited a strong light response but required a much brighter intensity of 85 mW/cm^2^ suggesting that sensitivity to DENAQ may also be disease-dependent. No DENAQ-driven light responses were observed in WT retinas when stimulated with medium-bright flashes. However, light responses kinetically similar to those driven by DENAQ were observed in four WT retinas (*retinas 7*, *8*, *10*, *11*) stimulated with the brightest (~85 mW/cm^2^) light intensity (Supplementary Figure S1). While such responses may be caused by the activation of melanopsin in intrinsically photosensitive RGCs (ipRGCs), the absence of responses at intensities expected to be nearly saturating for the melanopsin-driven ones suggests otherwise (Figure 1B). 

The dependence of parameters of the DENAQ-driven responses on light intensity is shown in Figure 1C. Brighter flashes resulted in increased per cell response amplitudes (counting all cells in a retina including light sensitive and insensitive ones, Figure 1C left), increased fraction of responding cells (Figure 1C middle) and increased averaged responses of individual cells (Figure 1C right). Four retinas producing strong DENAQ-driven responses (*retinas 1*, *3*, *4,* and *14*) had similar response-intensity dependence with responses appearing at intensities above 0.2 mW/cm^2^. In comparison light sensitivities of *retinas 5* and *6* were at least 10 times lower as demonstrated by the rightward shifts of their response–intensity curves (Figure 1C left). Note that short 0.1 s flashes were used to test response–intensity dependence in *retina 5* due to slow response recovery to longer flashes.

### 3.2. Kinetics of DENAQ-Driven RGC Firing in Degenerated Retinas 

**Figure 2:**
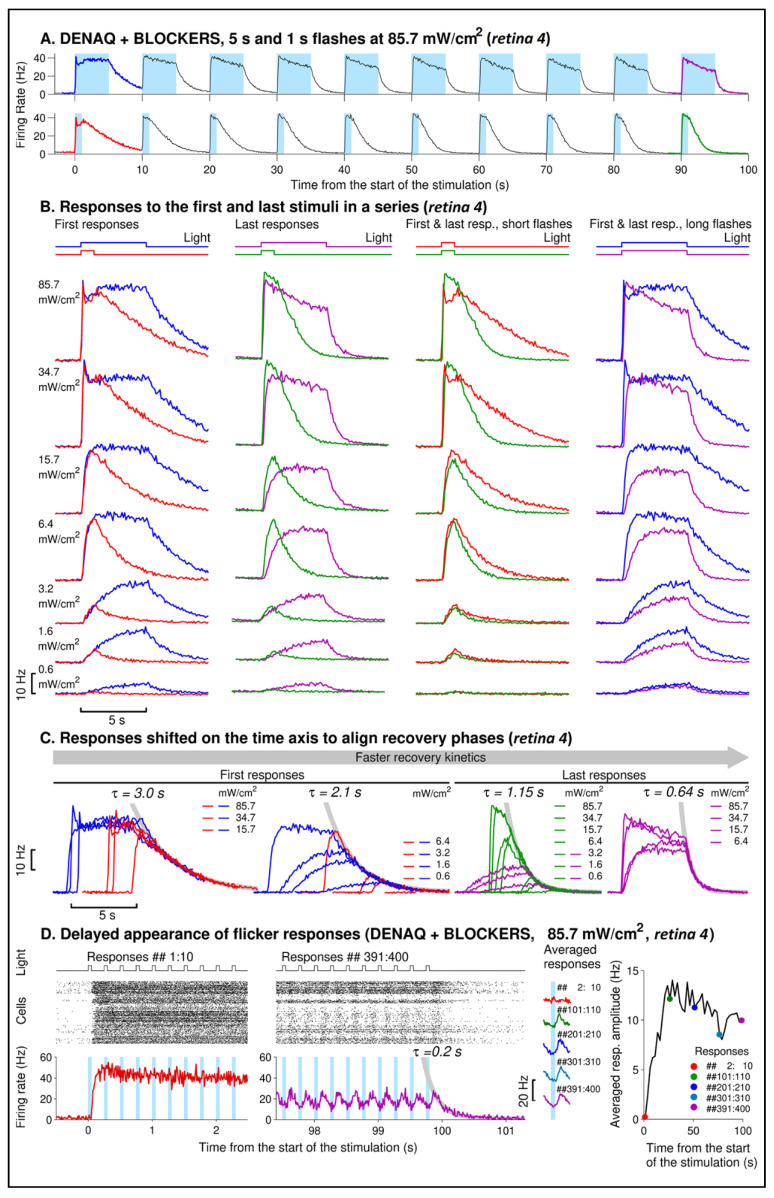
Repeated exposure to light leads to acceleration of response recovery and appearance of a flicker response. (**A**) Per cell averaged DENAQ-driven responses of *retina 4* (100 ms time bin) to ten brightest 5 s (upper trace) and 1 s (lower trace) flashes, the first and last responses in a series are shown in bold and color-coded, blue bars indicate delivery of light stimuli. Responses were obtained from a population of 179 light-sensitive cells identified under “DENAQ + BLOCKERS” conditions. (**B**) Comparison of first and last responses from stimulation series using 1 s and 5 s flashes of different intensities (each row of graphs presents responses to the flashes of the same intensity). Responses are colored according to the color code implemented on panel (**A**). Timing and duration of stimuli are indicated by the traces marked “Light” at the top of each column of graphs. (**C**) Responses from panel (**B**) shifted on the time axis to align their recovery phases. Recovery kinetics were fitted using single exponential decay traces (thick light gray traces, time constants indicated on the graphs). (**D**) Raster plots and averaged firing rate responses of the same 179 cells from *retina 4* to the first (1–10) and last (391–400) 10 flashes in the flicker stimulation series (50 ms flashes delivered at 4 Hz, total of 400 flashes in a series, firing rates were calculated using 10 ms time bin). Thick gray trace illustrates exponential decay of the firing rate at the end of the series. Traces labeled “Averaged responses” show examples of averaged responses to 10 consecutive flashes obtained at different moments during the flicker stimulation (excluding the response to the first flash), their amplitudes are provided on the rightmost graph (circles), the black trace connecting the symbols shows the averaged response amplitudes of consecutive groups of 10 consecutive responses. ##: corresponds to numbering of the responses.

As we observed that the response amplitude declined with repeated stimulations, most noticeably when stimulated with longer flashes at medium intensities (Figure 1A right), we further investigated the kinetics of DENAQ-driven light responses to assess its stability to repeated stimuli. We examined the first and last responses in the stimulation series consisting of ten short (1 s) or long (5 s) flashes delivered at 0.1 Hz resulting in 9 s or 5 s interflash intervals, respectively (Figure 2A). As expected, the rising phases of the first responses to the short and long flashes were nearly indistinguishable (Figure 2B, “First responses”), late responses to the longer flashes had slower activation kinetics (Figure 2B, “Last responses”). Activation phases of the first and last responses were again very similar for the short flashes (Figure 2B, “First & last resp., short flashes”). However late long-flash responses demonstrated slower activation at all intensities above 0.6 mW/cm^2^ and lower amplitudes at medium bright intensities (Figure 2B, “First & last resp., long flashes”). Note that the activation kinetics at the brightest intensity are too fast to reliably reveal the difference in activation phases of the first and last responses on the time scale used to plot traces in Figure 2B. In that case, responses were also inspected on a faster time scale when the difference in activation kinetics can be readily observed to avoid making the figure too complex). In addition, late flash responses had accelerated recovery kinetics which was especially noticeable for the longer flashes (Figure 2C). Similar effects (slower activation and accelerated recovery with repeated light exposure) were observed in all five retinas (*retinas 1*, *2*, *3*, *4*, *14*) which responses demonstrated sufficiently high signal-to-noise ratio and were suitable for this kind of analysis. However, the extent of the effect varied between retinas. For example, a slowdown in activation kinetics was observed for *retinas 1*, *2*, and *3* even when they were stimulated with short 1 s flashes. In addition, while shortening of the recovery time constant during 10-flash stimulation series was around 2.5–3 times for *retinas 2*, *4*, and *14*, it was shortened only by ~30% for *retina 1*, and by ~10% for *retina 3*. Resting retinas in the dark resulted in a partial recovery of the longer recovery kinetics, while the fastest kinetics were observed at the end of the flicker stimulation series. Indeed, the acceleration of the recovery with the repeated stimuli was likely responsible for the delayed appearance of a flicker response. For *retina 4* in particular, the fastest recovery observed at the end of the flicker series was characterized by the time constant as fast as 0.2 s, and stable responses to individual flashes were observed after around 100 flash repetitions (Figure 2D). 

DENAQ-induced flicker responses were reliably observed only at the brighter end of the intensity range (at and above 15 mW/cm^2^) when short (50 ms) flashes were bright enough to cause fast and sufficient depolarization triggering action potential firing. Of the three retinas tested at these conditions, *retinas 4* and *14* demonstrated accelerated recovery kinetics and both developed a flicker response. This was in contrast with *retina 1* which showed only moderate acceleration of recovery kinetics (the fastest time constant observed at the end of the flicker series was ~1 s). Consistently, while its firing rate was elevated during flicker stimulation, it was not able to resolve individual flashes in the flicker series. 

Individual cells demonstrated considerable variability in the kinetics of their responses. *Retina 4* cells can be (somehow subjectively) separated into four groups based on the shape of the responses they produce at the brighter end of the intensity range. Examples of group-averaged responses (red traces) and responses of individual cells (three cells per group) are shown in Figure S2. Out of 197 cells identified in the *retina 4*, 55 cells can be reliably assigned to one of the four groups, while responses of the remaining cells appear to be of mixed types demonstrating features attributable to more than one group. At the lower intensities (6 mW/cm^2^ and lower) activity of cells with “fast On-responses” and differences in the kinetics of all types of slow on-responses were greatly reduced. Note the considerable acceleration of response recovery towards the end of the stimulation series, consistent with the observations drawn previously from the analysis of the *retina 4* averaged responses.

### 3.3. MEA Analysis of Cone versus DENAQ-Driven RGC Firing

As was noted previously, *retina 4* was the only degenerated retina producing substantial cone-driven responses prior to DENAQ application, thus presenting an opportunity to investigate relations between cone- and DENAQ-driven RGC firing. Responses of this retina to flashes of four different intensities (standard and flicker series) recorded under three different perfusion conditions (perfusion with Ringer’s before DENAQ, perfusion with regular Ringer’s after DENAQ application, followed by Ringer’s containing synaptic blockers) are shown on Supplementary Figure S3. Application of DENAQ caused nearly complete disappearance of cone-driven responses at the dimmer intensities (3.2 mW/cm^2^; the dimmest intensity of 0.2 mW/cm^2^ was not used under “DENAQ/no BLOCKERS” condition) (panels B and E). At the brighter intensities, there were robust responses with amplitudes comparable to (15.7 mW/cm^2^, panels C and F) or exceeding (85.7 mW/cm^2^, panels D and G) corresponding cone-driven responses. DENAQ also caused a nearly complete disappearance of the well-defined cone-driven flicker response at all tested intensities, consistent with the slower recovery of DENAQ-driven vs. cone-driven responses (panels M and P, N and Q, O and R). Application of synaptic blockers resulted in a noticeably increased amplitude of the DENAQ-driven responses and delayed appearance of flicker response as was described earlier (panels E and I, F and J, G and K for the standard, and Q and U, R and V for the flicker series). However robust cone-driven responses observed at the dimmer intensities prior to the DENAQ application were either drastically reduced (panels A and H, standard stimulation series) or completely abolished (panels L and S, M and T, flicker series). The large difference in response kinetics observed under “pre DENAQ” and “DENAQ/no BLOCKERS” conditions (panels C and F, D and G), and the close similarity of the response kinetics under “DENAQ/no BLOCKERS” and “DENAQ + BLOCKERS” conditions (panels F and J, G and K) suggests that a DENAQ-driven component dominated the retinal response under “DENAQ/no BLOCKERS” conditions.

The higher light sensitivity of cone-driven responses compared to the DENAQ-driven ones is further shown by the traces presented in Supplementary Figure S4. Amplitude-intensity traces for the cone-drive responses were shifted leftward by more than one log10 unit and the peak intensities for cone-driven and DENAQ-driven responses were around 0.2 and 85 mW/cm^2^, respectively. Stimulation series at 3.2 mW/cm^2^ (1 s flashes) were delivered at the very start of the recording, likely before the retina had completely settled down in the MEA chamber. This may explain the appearance of the secondary peak at 3.2 mW/cm^2^ on the corresponding trace in Supplementary Figure S4 (note the absence of the secondary peak for the trace representing cone-driven responses to 5 s flashes).

Counting light-sensitive ganglion cells at intensities producing maximum cone-driven (0.2 mW/cm^2^, “pre DENAQ”) and maximum DENAQ-driven responses (85 mW/cm^2^, “DENAQ + BLOCKERS”) showed (Supplementary Figure S5) that out of 190 light-sensitive cells identified in degenerated *retina 4*, more than half had responses driven first by cones and then by DENAQ (96 cells). Slightly less than 50% of the cells (83 cells) did not show cone-driven responses but were strongly activated by light under “DENAQ + BLOCKERS” conditions, while a small number of cells (11 cells) demonstrating cone-driven responses were silenced after DENAQ application. Slightly less than half of the cells in the first two populations generated robust responses when perfused with Ringer’s solution without synaptic blockers following DENAQ application. These results suggest that the potentially beneficial contribution to vision of partially retained cone-driven function may be lost following DENAQ treatment in a degenerated retina.

### 3.4. DENAQ Confers Light Sensitivity to Mutant Canine Retinas following Intravitreal Injection

**Figure 3:**
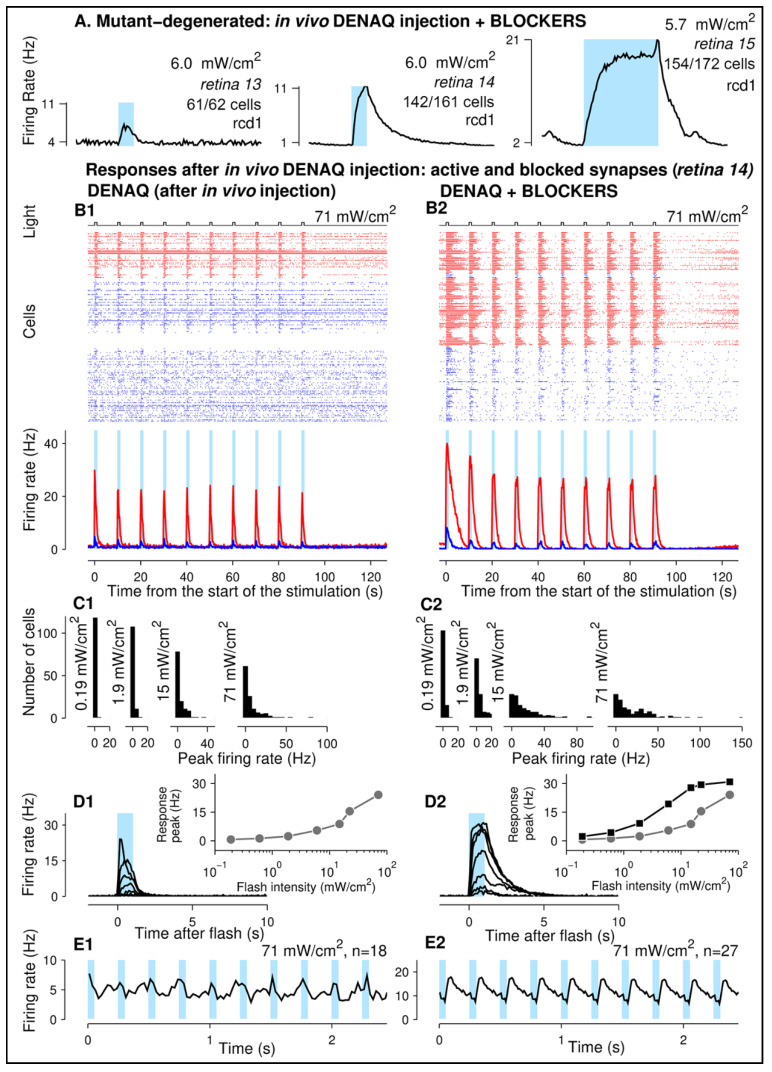
Responses elicited by in vivo DENAQ injection are similar to responses observed after ex vivo DENAQ application. Panel (**A**) shows averaged responses (per cell responses averaged for 10 flashes in a stimulation series) recorded from 3 degenerated retinas perfused with Ringer’s and synaptic blockers following in vivo DENAQ injection. Responses of cells producing less than 30 spikes per stimulation series were not counted. Number of cells per retina is given as number of cells used to calculate averaged response over total number of cells counted in the retina. Blue bars indicate light flashes. Results obtained from *retina 14* during perfusion with regular Ringer’s and with Ringer’s containing synaptic blockers following in vivo DENAQ injection are presented on panels (**B1**–**E1**) and panels (**B2**–**E2**), respectively (same axis scaling is used for all corresponding panels except panels (**E1**,**E2**), vertical scales are shown on the left of the figure). Raster plots and averaged per cell firing rates for 161 cells identified in *retina 14* are shown on panels (**B1**,**B2**), identical stimulation series were used under both perfusion conditions. Opposing lines on the raster graphs present data obtained from the same cells under the two conditions, red color indicates cells producing strong responses, and blue color indicates cells with reduced or abolished responses. Averaged firing rates were calculated separately for the “red” and “blue” cells and plotted using red and blue traces, respectively. Histograms on panels (**C1**,**C2**) give numbers of cells producing responses in particular range of amplitudes for different stimulation intensities (bar width is 5 Hz). Response families obtained from 32 cells producing robust light responses both before and after application of blockers are given on panels (**D1**,**D2**). Inserts show response peak amplitudes *vs* flash intensity data (amplitude-intensity curve from panel (**D1**) is re-plotted on panel (**D2**) for comparison). Panels (**E1**,**E2**) plot averaged flicker responses (light intensity and number of cells sensitive to flicker stimulation are given on the graphs, note different vertical scaling). To calculate averaged flicker response 400 responses in a stimulation series were separated into 40 consecutive groups, each group containing 10 consecutive responses, and responses from different groups were averaged for each of the two conditions. All firing rate traces except flicker responses were calculated using 100 ms time bin, flicker responses were calculated using 10 ms time bin. When present light blue bars indicate light flashes on all graphs.

To determine whether DENAQ can confer light sensitivity to RGCs following in vivo administration, intravitreal injections of the photoswitch (150 μL at 3 or 10 mM) were performed in three *PDE6B* mutant dogs (Supplementary Table S1), and retinas collected for MEA analysis 48 h later. Strong DENAQ-driven responses were observed in all three animals (Figure 3A). This included a 15-week-old dog (retina 15) with ongoing retinal degeneration (approx. 75% loss of rods) and two animals (*retinas 13* and *14*) at an age at which there is complete loss of rods.

The detailed examination of responses recorded from *retina 14* is presented in Figure 3(B1–E2). Consistent with results observed during ex vivo DENAQ application (Supplementary Figure S5), inhibition of synaptic transmission in DENAQ-treated retina substantially increased the number of light-sensitive cells while only a few cells were silenced by DENAQ treatment (Figure 3(B1,B2)). The number of responsive cells and amplitudes of their responses increased with flash intensity under both conditions (Figure 3(C1,C2)). For a subpopulation of 32 cells that were light sensitive both before and after perfusion with synaptic blockers, maximum response amplitudes before and after blockers were similar but light sensitivity in blockers was about 10 times higher (Figure 3D1,D2). Finally, under both conditions, a flicker response was detected at the brightest intensity, with a larger number of responding cells and more clearly defined responses observed during perfusion with synaptic blockers (Figure 3(E1,E2)).

### 3.5. P2X Receptors and HCN Channels Are Upregulated in Degenerated Canine Retinas

**Figure 4:**
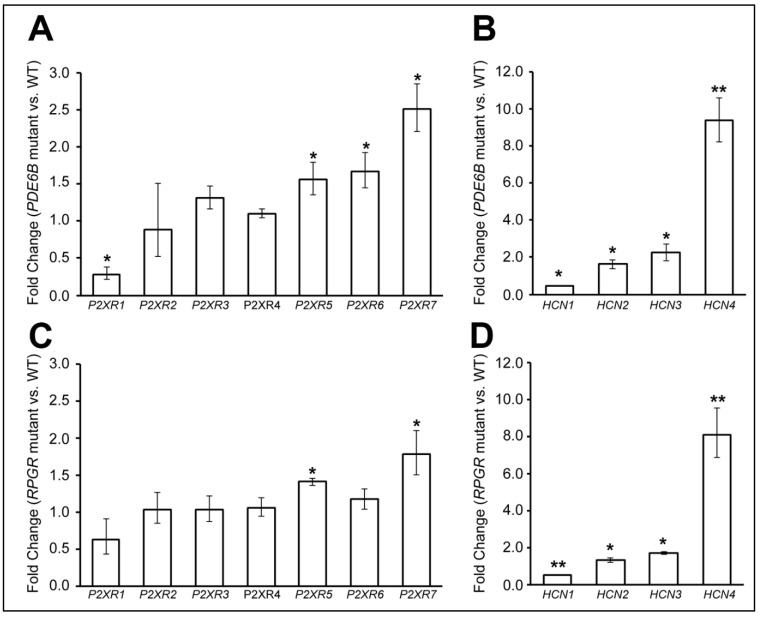
Changes in mRNA expression of P2X receptors and HCN channels in mutant canine retinas with advanced photoreceptor loss. Quantitative PCR analysis of the changes in expression levels of P2X receptors and HCN channels in PDE6B mutants (**A**,**B**) and RPGR mutants (**C**,**D**) when compared to levels in normal retinas. The error bars represent the range of fold change values on *n* = 3 animals per group. * *p*-value ≤ 0.05; ** *p*-value ≤ 0.001.

In murine models of retinitis pigmentosa, a degeneration-dependent increase in expression of ATP-gated purinergic (P2X) receptors [22] has been proposed as the mechanism of entry of DENAQ into RGCs enabling this cationic photoswitch to bind to hyperpolarization-activated cyclic-nucleotide gated channels (HCN) and control sodium influx [13]. To determine whether similar changes occur in dogs undergoing retinal degeneration, we examined by qRT-PCR the levels of expression of seven P2X receptor subtypes (P2XR 1–7) and four HCN channels (HCN 1–4) in the retinas of *PDE6B* and *RPGR* mutant dogs at an advanced stage of disease (>50% photoreceptor loss) and compared them to levels in WT dogs. Significantly increased levels of *P2XR5*, *P2XR6*, *P2XR7*, *HCN2*, *HCN3*, and *HCN4* transcripts were found in *PDE6B* mutant retinas, and *P2XR5*, *P2XR7*, *HCN2*, *HCN3*, and *HCN4* transcripts in *RPGR* mutant retinas, while levels of P2XR1 and HCN1 were reduced in both diseases (Figure 4). These results suggest that degenerated canine and murine retinas share a potentially similar mechanism of entry and action of DENAQ into RGCs.

### 3.6. Dose-Dependent Focal Retinal Toxicity following Intravitreal Injection of DENAQ

**Figure 5:**
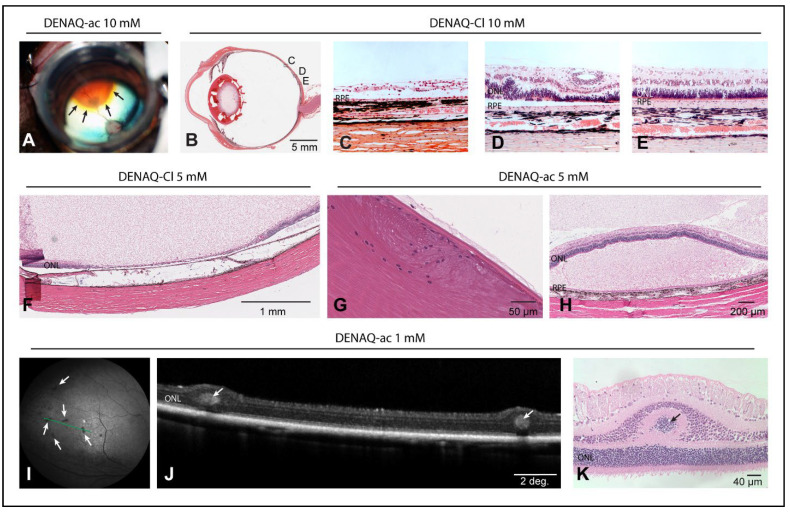
Ocular lesions following intravitreal injection of DENAQ. (**A**) Peri-operative photography showing limited miscibility of 150 μL of DENAQ-acetate (10 mM) (see black arrows) in the vitreous gel of a WT dog (2135-OS) following bolus injection. (**B**) Photomicrograph of a sagittal histological (H&E) section of a WT canine globe (2134-OD) injected one week prior with 150 μL of DENAQ-chloride (10 mM), indicating the location of the magnified views shown in panels C,D. (**C**) Focal area of complete retinal atrophy. (**D**) Focal area of disrupted retinal lamination. (**E**) Illustration of an area of preserved retinal structure. (**F**) Focal area of complete retinal atrophy in a WT canine globe (WM14-OD) injected one week prior with 150 μL of DENAQ-chloride (5 mM) in 50% DMSO (**G**,**H**) Posterior cortical cataract (**G**) and exudative retinal detachment (**H**) in the contralateral eye (WM14-OS) injected one week prior with 150 μL of DENAQ-acetate (5 mM) in 10% DMSO. (**I**–**K**) Focal retinal folds in a WT canine globe (E1063-OS) injected one week prior with 150 μL of DENAQ-acetate (1 mM) are detectable as dark multifocal spots (white arrows) by confocal scanning laser ophthalmoscopy (**I**) and correspond to photoreceptor rosettes seen by optical coherence tomography (**J**) and histology (**K**).

To begin assessing the short-term ocular tolerance of DENAQ in a human-sized eye, WT and *PDE6B* mutant dogs were injected with a 2-log range of concentrations (0.1–10 mM) of DENAQ dissolved in a solution of DMSO/BSS (Supplementary Table S2) and followed clinically for 1 to 2 weeks post-injection. Dogs were then euthanatized, and eyes were collected for histological examination. Following intravitreal injection there was poor miscibility of the DENAQ with the vitreous gel, resulting in focal accumulation of the orange-colored photoswitch solution (Figure 5A). Features of DENAQ-induced ocular toxicity that were detectable by ophthalmic examination and/or histological assessment are summarized in Table 1. Most serious adverse effects were seen in eyes injected with the higher concentrations (1–10 mM) and included impaired visually guided navigational skills, serous retinal detachment (Figure 5H), and focal retinal lesions of atrophy, disrupted lamination, and/or retinal folds (Figure 5B–F,I–K). Posterior subcapsular cataracts (Figure 5G) were found in eyes treated with DENAQ concentrations as low as 0.3 mM. In eyes injected with the lowest (0.1 mM) concentration, there was mild ocular discomfort and transient anterior uveitis (aqueous flare) which was also seen in the vehicle control group. Taken together, these observations suggest that poor miscibility of DENAQ upon injection into the vitreous gel results in a localized high concentration of the photoswitch that can cause focal toxicity to the underlying retinal area. 

### 3.7. Delivery of DENAQ in Vitrectomized Eyes Expands the Ocular Tolerance Range

**Figure 6:**
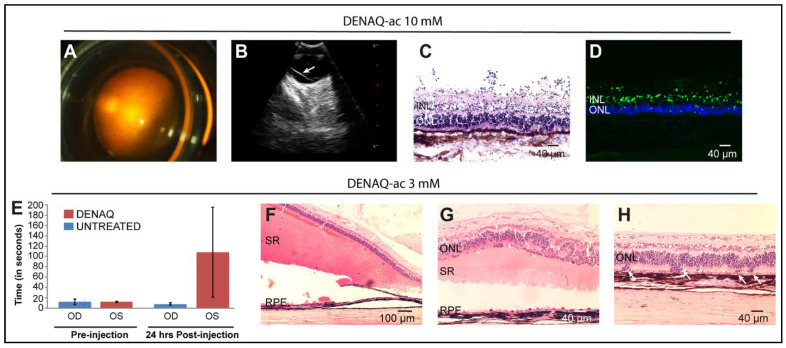
Ocular lesions following injection of DENAQ into the vitreal cavity of vitrectomized eyes. (**A**) Peri-operative photography showing widespread diffusion throughout the retinal surface of 150 μL of DENAQ-acetate (10 mM) following bolus injection in a *PDE6B* mutant dog (AS379-OS). (**B**) B-scan ultrasound showing retinal detachment (white arrow) in the eye of a *PDE6B* mutant dog (2202-OD) injected 6 days prior with 150 μL of DENAQ-acetate (10 mM). (**C**) H&E stained retinal cross-section of a WT dog (WM12-OD) showing severe necrosis of the inner retina and disrupted ONL lamination. (**D**) Retinal section from the same eye as in (**C**) showing TUNEL-positive labeling (in green) within the INL and ONL, 24 h after injection of 150 μL of DENAQ-acetate (10 mM). (**E**) Transit time in an obstacle avoidance course under 65 Lux of ambient white light before and 24 h after injection of 150 μL of DENAQ-acetate (3 mM) in the left eye (OS) of a PDE6B mutant dog (2081). (**F**,**G**) Subretinal hemorrhage, retinal detachment, and disrupted retinal lamination in the eye of a WT dog (CFFCAZ-OD) injected one week prior with 150 μL of DENAQ-acetate (3 mM). (**H**) Focal disruption of the external limiting membrane with migration of photoreceptor cell bodies (white arrows) within the subretinal space of another WT dog (CFFCKM-OS) injected one week prior with 150 μL of DENAQ-acetate (3 mM).

In an attempt to circumvent the occurrence of focal retinal toxicity caused by localized high concentrations of DENAQ, we injected a range of DENAQ concentrations (1–10 mM in DMSO/BSS) in eyes of WT and mutant dogs after vitrectomy (Supplementary Table S2). Peri-operative imaging of the injection showed a much-improved distribution of the photoswitch solution throughout the retinal surface (Figure 6A), although miscibility with the aqueous humor present in the vitreal cavity was not immediately seen (Supplementary Video S1). Features of DENAQ-induced ocular toxicity that were detectable by ophthalmic examination and/or histological assessment are summarized in Table 2. All 3 dogs injected with the highest (10 mM) concentration of DENAQ had a retinal detachment detected as early as one-day post-injection and confirmed by ultrasound (Figure 6B). Histological examination of the retina of one of these dogs that were terminated one-day post-injection showed fulminant necrosis of the inner retina characterized by a near complete loss of the nerve fiber layer, ganglion cell layer, and inner plexiform layer, and a disrupted lamination of the inner nuclear layer (INL) and outer nuclear layer (ONL) (Figure 6C). TUNEL-positive labeling was predominantly seen in the INL with rare features of cell death found in the ONL (Figure 6D). Visual impairment in DENAQ-treated eyes was seen as early as 24 h post-injection in some of the dogs in the 10 mM and 3 mM groups (Figure 6E; Supplementary Video S2). The lowest concentration at which retinal alterations including subretinal hemorrhage, retinal detachment, and disrupted retinal lamination were still seen histologically (Figure 6F–H) was 3 mM. Eyes treated with 1 mM DENAQ did not show any significant signs of toxicity other than some transient anterior uveitis and ocular discomfort that could not be unequivocally attributed to the photoswitch compound.

## 4. Discussion

Developing a photopharmacological approach aimed at restoring vision for a variety of outer retinal degenerative diseases including AMD, inherited retinal dystrophies, and retinal detachments will require meticulous validation in diverse model systems before considering translation to the clinic. Our efforts at evaluating the efficacy of DENAQ, an azobenzene-based photoswitch, in non-allelic canine models of late-stage retinal degeneration confirm the potential of this strategy at bestowing light sensitivity to RGCs. However, unexpected ocular toxicity observed in vivo at doses that confer RGC photosensitization will limit the clinical use of this specific photoswitch. 

### 4.1. DENAQ Drives Light Responses in the Degenerated Canine Retinas

DENAQ treatment conferred light sensitivity to RGCs in the degenerated canine retinas (Figure 1). Two main observations strongly suggest that responses in the treated degenerated retinas were DENAQ-driven: (1) all but one of the degenerated retinas that subsequently responded to DENAQ had only very weak or abolished light responses prior to DENAQ application, and (2) following DENAQ treatment strong light responses were observed from the retinas in which synaptic transmission to the RGCs was blocked. Responses kinetically similar to those driven by DENAQ were also observed in four out of six WT retinas perfused with synaptic blockers following DENAQ application, albeit only at the brightest flash intensity (85 mW/cm^2^, Supplementary Figure S1). It seems unlikely that these responses were caused by the melanopsin excitation because no responses were evoked by the dimmer light (3 mW/cm^2^, Figure 1B, right column panels) at intensities when amplitudes of melanopsin-driven responses are supposed to be saturated [23,24,25]. The much lower sensitivity of presumably DENAQ-driven responses observed in some of the treated WT retinas suggests that, compared to the degenerated retinas, they are affected by the DENAQ treatment to a much lesser extent.

These observations were similar to previously reported studies in mice, which showed DENAQ-induced photosensitization only in degenerated retinas [9]. Yet, the results of our current study show that in dogs with ongoing photoreceptor degeneration including some with persistent cone-mediated function, DENAQ can still confer light sensitivity to RGCs, suggesting that reaching end-stage photoreceptor degeneration is not a prerequisite for using photoswitches. In addition, contrary to that reported in WT and non-functional triple KO murine retinas with structurally intact rods and cones, photosensitization of RGCs was also found in the WT canine retina, yet this required stimulation with the brightest light intensity. Based on the kinetics and intensity dependence of those responses, we have attributed this to a DENAQ-driven process rather than to the direct activation of ipRGCs. The molecular basis for the degeneration-specific increased sensitivity of RGCs to azobenzene photoswitches is thought to involve two synergistic processes that are potentiated by the release of retinoid acid (RA) and ATP. Rod and cone loss leads to increased free retinaldehyde, which is converted into RA by the enzyme RALDH. RA through its receptor RAR is a regulator of gene transcription, and some of the upregulated genes in RGCs are P2X receptors and HCN channels [13,26]. Once activated by extracellular ATP that is released by dying photoreceptors, the P2X receptors which are large cationic pores increase the membrane permeability of RGCs thus enabling the entry of DENAQ and its binding to its molecular target, the HCN channels [9,27]. Consistent with results reported in rd1 (*PDE6B* mutant) mouse retinas [13], we observed a significant upregulation of *P2XR7*, *HCN3*, and *HCN4* mRNA in degenerated retinas from two non-allelic canine models (rcd1/*PDE6B*, and xlpra2/*RPGR*). No changes in *P2XR4* expression were found, however, levels of *P2XR5*, which expresses in RGCs and in the INL, were upregulated in both models. The significant decrease in *HCN1* levels was consistent with its specific expression in the photoreceptors [28], and the significant loss of this cell population at the selected stage of disease. 

Following ex vivo DENAQ application or in vivo DENAQ injection, photoswitch-driven light responses in degenerated retinas perfused with synaptic blockers were observed at intensity levels that can be encountered under natural conditions (as low as 0.2 mW/cm^2^, Figure 1C). Amplitudes of the DENAQ-driven response increased with light intensity due to the higher number of responding cells (up to 50%−90% of cells responding at the brightest intensity) and higher per cell response.

Taken together, these results further suggest that photosensitization of RGCs to DENAQ requires disease-induced alterations in RGC membrane permeability to ions and to the azobenzene photoswitch.

### 4.2. Repeated Light Stimuli Slow Activation and Accelerate Recovery of the DENAQ-Driven Responses

Repeated light stimuli resulted in slower activation of the DENAQ-driven responses (Figure 2B), reduced response amplitudes (most noticeable at the medium bright intensities) and accelerated response recovery (Figure 2C). These effects might be caused by the same mechanisms which are involved in spike firing adaptation, such as opening of the slow Ca^2+^-activated K^+^-channels (decay time of around 4–8 s) by Ca^2+^ ions entering cells during action potential firing [29]. The resulting hyperpolarizing potassium currents would reduce DENAQ-dependent depolarization potentially leading to a slower activation, reduced steady-state firing, and faster recovery after flash. However, at the brightest intensities, DENAQ-induced depolarization may be strong enough to overcome hyperpolarizing currents forcing cells to maintain high firing rates resulting in a more stable response amplitude. Slower activation could also be caused by the slow recovery of the DENAQ-driven channel, leading to the reduced number of channels available for activation after repeated stimuli. Response kinetics could be also affected by the time and light-dependent suppression of the firing rate demonstrated by some cells (responses of the last two groups in Figure S2), although at this point, we do not have data to speculate about its molecular mechanisms. The acceleration of the response recovery with the repeated stimuli appears to be the main cause of the gradual appearance of a flicker response under “DENAQ + BLOCKERS” conditions (Figure 2D). Flicker responses were observed at the brighter end of the intensity range, likely because both activation and inactivation kinetics were sufficiently fast under those conditions. Since the ability of DENAQ-treated retinas to resolve faster changes in light improves with increasing light intensity, the use of image intensifiers [30] might prove beneficial after potential DENAQ treatment. 

### 4.3. DENAQ can Override Responses of the Surviving Cones in the Degenerated Retinas

An important clinically relevant question is determining the effect of DENAQ in a degenerated retina that still has retained partial cone function. Results obtained from one such retina (*retina 4*, Supplementary Figures S3 and S4) showed that while DENAQ caused a noticeable increase in the response amplitude at the brightest end of the intensity range, responses at the dimmer intensities were greatly reduced or abolished. This was especially pronounced for the responses recorded under clinically relevant conditions during perfusion with Ringer’s solution following DENAQ treatment but before the application of synaptic blockers. Under these conditions, DENAQ also caused the complete disappearance of robust cone-driven flicker response at all tested intensities. Both non-responding RGCs and RGCs generating cone-driven responses under “pre DENAQ” conditions appear to be switched to the generation of DENAQ-driven responses following the DENAQ application (Supplementary Figure S5). Some improvement in the bright flash responses at the cost of considerably lower light sensitivity and a loss of a flicker response would likely have a negative effect on visual function. Such a possibility should be given thorough consideration for any potential treatment of patients who did not reach the final stages of retinal degeneration.

### 4.4. Switching off Retinal Synaptic Transmission Enhances DENAQ-Driven Response

DENAQ-driven responses were observed prior to the perfusion with synaptic blockers both after ex vivo DENAQ application (Supplementary Figures S3 and S5) and after in vivo DENAQ injection (Figure 3). We noted, however, that blocking retinal synaptic transmission resulted in a substantial increase in the response amplitude and in the number of responding cells (likewise blocking of synaptic transmission resulted in better-defined flicker responses). This suggests that DENAQ may also have an effect on upstream retinal neurons that provide inhibiting inputs to the RGCs. Translation to the clinic of a photoswitch such as DENAQ may therefore require its co-delivery with pharmacological approaches that isolate RGCs from the remodeled retinal neurocircuitry. 

### 4.5. Efficiency of DENAQ Treatment

Smaller DENAQ-driven responses were observed in the older mutant retinas suggesting that efficiency of treatment may depend on the animal’s age/stage of disease (Figure 1 *retina 5*). Following the initial degeneration of photoreceptor cells, progressive neuro-remodeling of the inner retina with gradual loss of second order neurons and RGCs ultimately results in complete retinal atrophy [31,32,33]. Thus, while expected to be wide, the optimal time window for photoswitch intervention needs to be well defined. Based on these preliminary preclinical results, one would envision that a photochemical compound that targets primarily RGCs could be delivered as soon as photoreceptor-driven responses are no longer detectable and until functional RGCs are still present. Developing adequate non-invasive tools to monitor the structural and functional integrity of RGCs in late-stage retinal degenerative diseases is needed [34]. 

### 4.6. DENAQ-Associated Ocular Toxicity

Dose-dependent ocular toxicity that included clinical signs of uveitis, posterior subcapsular cataracts, retinal detachment, retinal atrophy, acute visual impairment, and glaucoma was observed following intravitreal injection of 150 μL of DENAQ at concentrations ranging from 0.3 mM to 10 mM. In non-vitrectomized eyes, whose dense vitreous limited the miscibility of the injected DENAQ solution, this resulted in a high local dose of the photoswitch that was focally retinotoxic. An improved safety profile was achieved in vitrectomized eyes as DENAQ dispersed over a larger surface of the retina. In these eyes, signs of toxicity were detected with DENAQ concentrations starting at 3 mM. Acute necrosis of the inner retina with cell death in the INL (Figure 6C,D), associated with disruption of the external limiting membrane (Figure 6H) suggests a potential involvement of Müller glial cells in the mechanism of DENAQ-induced retinal toxicity. Müller cells span through the entire neuroretina from the external limiting membrane to the vitreoretinal surface and play a key role in transcellular water transport and spatial potassium buffering [35]. As Müller cells express the P2X7 receptor [36] this may have resulted in the acute entry of DENAQ, impairment of potassium efflux by altering the activity of rectifying Kir4.1 potassium channels, cytotoxic swelling, and inner retinal degeneration [37]. 

The aggregation of DENAQ likely results in a highly concentrated dose that is retinotoxic. The use in mice of a slow-release formulation containing an FDA-approved derivative of beta-cyclodextrin (Captisol^®^) to encapsulate a third-generation photoswitch (BENAQ), has been recently shown to eliminate focal aggregates, increase solubility, and enable pan-retinal diffusion of the photoswitch for several weeks [38]. Regardless of the precise mechanism of DENAQ-induced retinotoxicity, it is noteworthy to mention that similar acute ocular toxicity was seen in dogs intravitreally injected with AAQ, a first-generation azobenzene photoswitch (Beltran, personal communication). Importantly, no toxicity was observed with either AAQ (1 μL at 80 mM) or DENAQ (2 μL at 5 mM) injected intravitreally in murine eyes [8,9]. Whether species specificity, dosage, or anatomical (e.g., vitreous consistency) differences account for the absence of toxicity seen in rodents is currently unknown but highlights the importance of assessing the ocular tolerance of novel drugs in non-rodent species, particularly those that have human-sized eyes before moving to a potential clinical trial.

In summary, we report the testing of an azobenzene-derived photoswitch in a large animal model of inherited retinal degeneration with advanced photoreceptor loss. Our results show that photosensitization of RGCs with DENAQ is enhanced in degenerated- retinas, but also show that the potential contribution of surviving cones to RGC firing is overridden by the photoswitch. As patients affected with AMD, or retinitis pigmentosa frequently retain regional cone-mediated function even in the advanced stage of disease, it will be critical to assess the risk-benefit of such photopharmacological intervention. Finally, DENAQ’s narrow therapeutic index warrants further screening of more potent next-generation photoswitches (e.g., BENAQ) [11] that can offer a long-lasting effect at lower doses and with an improved safety profile. 

## Figures and Tables

**Table 1:** Clinical and histological findings of ocular toxicity associated with intravitreal injection of DENAQ in non-vitrectomized WT and *PDE6B* mutant canine eyes.

DENAQ Concentration	Visual Impairment	Ocular Discomfort	Anterior Uveitis	Glaucoma	Posterior Subcapsular Cataract	Posterior Uveitis	Retinal Detachment	Retinal Atrophy	Disrupted Retinal Lamination	Retinal Folds
10 mM	2/4	2/4	4/4	1/4	4/4	2/4	1/4	2/4	2/4	0/4
5 mM	6/6 *	0/6	6/6	0/6	5/6	5/6	5/6	3/6	2/6	0/6
1 mM	4/4	0/4	4/4	0/4	2/4	0/4	1/4 **	0/4	0/4	2/4
0.3 mM	0/3	1/3	2/3	0/3	2/3	1/3	0/3	0/3	0/3	0/3
0.1 mM	0/3	1/3	2/3	0/3	0/3	0/3	0/3	0/3	0/3	0/3
Vehicle ***	0/2	0/2	2/2	0/2	0/2	0/2	0/2	0/2	0/2	0/2

*: Includes both eyes of a 15-week-old *PDE6B* mutant dog that had persistent cone-mediated vision before DENAQ injection. **: Limited to the canine fovea-like area. ***: 50% DMSO in BSS.

**Table 2:** Clinical and histological findings of ocular toxicity associated with intravitreal injection of DENAQ in vitrectomized WT and *PDE6B* mutant canine eyes.

DENAQ Concentration	Visual Impairment	Ocular Discomfort	Anterior Uveitis	Vitreal Hemorrhage	Retinal Detachment	Retinal Atrophy	Disrupted Retinal Lamination
10 mM	1/3	3/3	3/3	0/3	3/3	1/2	1/2
3 mM	2/3 *	2/3	3/3	1/3	1/3	0/2	2/2
1 mM	0/3	1/3	2/3	0/3	0/3	0/3	0/3
Vehicle **	0/1	0/1	0/1	0/1	0/1	0/1	0/1

*: Includes the eye of a 261-week-old *PDE6B* mutant dog that had persistent cone-mediated vision before DENAQ injection and worse function than the contralateral (uninjected) eye. **: 3% DMSO in BSS.

## Supplementary Materials

The following supporting information can be downloaded at: https://www.mdpi.com/article/10.3390/pharmaceutics14122711/s1. **Supplementary Figure S1.** Small light responses observed in 4 WT retinas under brightest stimulation following DENAQ application. Averaged per cell per flash responses from 4 WT retinas stimulated with the brightest 5 s flashes during perfusion with synaptic blockers following ex vivo DENAQ application. Light blue bars indicate time and durations of light stimuli. Cells producing less than 30 spike per stimulation series (total series duration 150 s) were excluded from the analysis. Number of cells per retina is given as number of cells used to calculate averaged retinal response over total number of cells identified in the retina. **Supplementary Figure S2**. Responses of individual cells show variable activation and recovery kinetics. A total of 55 cells from *retina 4* were separated into 4 groups based on the shape of their light responses. Rows of panels present group averaged (red traces) and individual (black traces) cell responses. Number of cells in each group is given on the left of the corresponding group-averaged traces. Three examples of individual cell responses are shown per group. The two columns of panels on the left show responses to the first and last flashes in the stimulation series containing 10 one-second flashes. End of series responses were calculated by averaging responses to flashes numbering 8, 9 and 10. The next two columns show initial and end of series responses for light series containing 10 five-second flashes (see notes above each column). Traces were calculated using 100 ms time bin. Light blue bars indicate light stimulation. Horizontal and vertical scale bars are provided on the figure. **Supplementary Figure S3**. Remaining cone function mainly disappears following DENAQ application. The figure shows responses of *retina 4* to flashes of four different intensities, recorded using standard and flicker stimulation series under different perfusion conditions. The left column (panels A,B,C,D,L,M,N,O) shows pre-DENAQ responses, middle column (panels E,F,G,P,Q,R) shows responses obtained during perfusion with Ringer’s solution following DENAQ application (“DENAQ/no BLOCKERS”, only 3 brighter flight intensities were used under these conditions), and the right column (panels H,I,J,K,S,T,U,V) shows responses obtained during perfusion with Ringer’s solution containing synaptic blockers (“DENAQ + BLOCKERS”). The upper half of the figure shows per cell averaged responses to a standard stimulation series (10 one-second flashes delivered at 0.1 Hz, panels A through K). Each of the four rows shows responses to the flashes of a particular intensity as indicated to the left of the graphs, recorded under three different perfusion conditions. In a similar way the lower half of the figure (panels L through V) shows per cell per flash averaged responses to the flashes of the same four intensities delivered during flicker stimulation series (400 flashes at 4 Hz, 50 ms flash duration) under the same three perfusion conditions. To document dynamic changes in the flicker response, each panel (corresponding to a particular flicker intensity and perfusion condition) shows 5 responses obtained by averaging responses ## 1:10, 101:110, 201:210, 301:310 and 391:400 as indicated on the graph. Colored bars indicate light stimulation events. Black dotted lines below the traces indicate zero-firing rate levels. Responses to standard and flicker series were calculated using 100 ms and 10 ms time bins, respectively. Cells producing less than 30 spikes per stimulation series (150 s) were excluded from the analysis. For clarity purposes, gray dotted lines were used to connect the first two averaged responses on panels U and V, and the trace on panel B was vertically offset down by 10 Hz to separate it from the trace on panel A. **Supplementary Figure S4**. Cone-driven responses in degenerated retina reach maximum amplitude at lower light intensities compared to DENAQ-driven responses. Plots show amplitudes of the *retina 4* averaged per cell per flash responses to standard stimulation series under different perfusion conditions as indicated on the graph. Cone responses were acquired during perfusion with Ringer’s before DENAQ application and response peak amplitudes were measured at 150 ms after the flash onset. DENAQ-driven responses were recorded during perfusion with Ringer’s and synaptic blockers following ex vivo DENAQ application, response amplitudes were measured at the flash offset (traces for “DENAQ + BLOCKERS” condition replot *retina 4* response-intensity dependencies from the left panel of Figure 1C). **Supplementary Figure S5**. DENAQ overrides cone-driven pathway in degenerating retinas. Raster plots and spike firing rates were obtained from three populations of cells identified in a retina demonstrating cone-driven responses prior to the DENAQ treatment (*retina 4*). A total of 96 cells in the 1st population responded both before (“pre DENAQ”) and after DENAQ application when retina was perfused with Ringers containing synaptic blockers (“DENAQ + BLOCKERS”). A total of 83 cells in the 2nd population responded under “DENAQ + BLOCKERS” but not “pre DENAQ” conditions and 11 cells in the 3rd population responded under “pre DENAQ” but not “DENAQ + BLOCKERS” conditions. The first two populations were further subdivided into two sub-populations each to identify responding and non-responding cells when retina was perfused with plain Ringers following DENAQ application (“DENAQ/no BLOCKERS”). Cells not responding under any conditions were excluded from the analysis. Note that intensities used for “pre DENAQ” and post DENAQ (“DENAQ/no BLOCKERS”, “DENAQ + BLOCKERS”) conditions were selected to produce maximum cone- and DENAQ-driven responses and are different (0.2 and 85 mW/cm^2^, respectively). The order of plots from left to right represents regular experimental protocol: DENAQ application followed by perfusion with Ringers, which was in turn followed by perfusion with Ringers with synaptic blockers. **Supplementary Table S1**. List of retinas and experimental conditions used for MEA studies. **Supplementary Table S2**. List of animals and experimental conditions used for ocular tolerance studies. **Supplementary Table S3**. List of dog retinas used for qRT-PCR studies. **Supplementary Table S4**. List of primers used for qRT-PCR. **Supplementary Video S1**. Injection of a solution of DENAQ in a vitrectomized eye. **Supplementary Video S2**. Obstacle avoidance course testing of the visually guided behavior of a mutant *PDE6B* dog before and 24 h after injection of DENAQ (3 mM) in a vitrectomized eye.
