# Supplementary material for: Photochemical Restoration of Light Sensitivity in the Degenerated Canine Retina
Source: Pharmaceutics. 2022 Dec 3;14(12):2711. doi: 10.3390/pharmaceutics14122711 (PMC9783220; doi:10.3390/pharmaceutics14122711)
Supplement: Supplementary file 1 [file pharmaceutics-14-02711-s001.zip › Supplementary Table S2 List of animals and conditions used for ocular tolerance studies.pdf]

Table S2: List of animals and experimental conditions used for ocular tolerance studies.

	Dog ID-Eye	Status	Sex	Age at injection (weeks)	Compound	Concentration (mM)	Volume (μL)	vehicle	Inject-term time interval
**No vitrectomy:**									
	2134-OD	WT	F	36	DENAQ-Cl	10	150	50% DMSO in BSS	1 week
	2135-OD	WT	M	53	DENAQ-Cl	10	150	50% DMSO in BSS	2 weeks
	2139-OD	*PDE6B* mutant	M	34	DENAQ-Cl	10	150	50% DMSO in BSS	1 week
	2135-OS	WT	M	53	DENAQ-ac.	10	150	10% DMSO in BSS	2 weeks
	WM10-OD	WT	M	15	DENAQ-Cl	5	150	50% DMSO in BSS	1 week
	WM14-OD	WT	F	15	DENAQ-Cl	5	150	50% DMSO in BSS	1 week
	WM9-OD	*PDE6B* mutant	M	15	DENAQ-Cl	5	150	50% DMSO in BSS	1 week
	WM10-OS	WT	M	15	DENAQ-ac.	5	150	10% DMSO in BSS	1 week
	WM14-OS	WT	F	15	DENAQ-ac.	5	150	10% DMSO in BSS	1 week
	WM9-OS	*PDE6B* mutant	M	15	DENAQ-ac.	5	150	10% DMSO in BSS	1 week
	E1063-OD	WT	M	16	DENAQ-Cl	1	150	50% DMSO in BSS	1 week
	E1065-OD	WT	M	16	DENAQ-Cl	1	150	50% DMSO in BSS	1 week
	E1063-OS	WT	M	16	DENAQ-ac	1	150	10% DMSO in BSS	1 week
	E1065-OS	WT	M	16	DENAQ-ac	1	150	10% DMSO in BSS	1 week
	M705-OD	WT	M	43	DENAQ-ac	0.3	150	10% DMSO in BSS	1 week
	M706-OD	WT	F	41	DENAQ-ac	0.3	150	10% DMSO in BSS	1 week
	RC662-OD	WT	F	207	DENAQ-ac	0.3	150	0.1% DMSO in BSS	1 week
	M705-OS	WT	M	43	DENAQ-ac	0.1	150	10% DMSO in BSS	1 week
	M706-OS	WT	F	41	DENAQ-ac	0.1	150	10% DMSO in BSS	1 week
	RC662-OS	WT	F	207	DENAQ-ac	0.1	150	0.1% DMSO in BSS	1 week
	2134-OS	WT	F	36	vehicle	/	150	50% DMSO in BSS	1 week
	2139-OS	*PDE6B* mutant	M	34	vehicle	/	150	50% DMSO in BSS	2 weeks
**With vitrectomy:**									
	WM12-OD	WT	F	231	DENAQ-ac	10	150	10% DMSO in BSS	1 day
	AS379-OS	*PDE6B* mutant	F	56	DENAQ-ac	10	150	10% DMSO in BSS	2 days
	2202-OD	*PDE6B* mutant	F	128	DENAQ-ac	10	150	10% DMSO in BSS	1 week
	2081-OS	*PDE6B* mutant	F	261	DENAQ-ac	3	150	10% DMSO in BSS	2 days
	CFFCAZ-OD	WT	F	41	DENAQ-ac	3	150	3% DMSO in BSS	1 week
	CFFCKM-OS	WT	F	40	DENAQ-ac	3	150	3% DMSO in BSS	1 week
	WM12-OS	WT	F	231	DENAQ-ac	1	150	10% DMSO in BSS	1 day
	CFFCBE-OS	WT	F	38	DENAQ-ac	1	150	1% DMSO in BSS	1 week
	CFFCBE-OD	WT	F	37	DENAQ-ac	1	150	1% DMSO in BSS	2 weeks
	IG71-OS	WT	M	268	vehicle	/	150	3% DMSO in BSS	1 week

Abbreviations OD: right eye; OS: left eye; WT: wildtype; F: female; M: male; DENAQ-Cl: DENAQ-chloride; DENAQ-ac: DENAQ-acetate; DMSO: dimethyl sulfoxide; BSS: balanced salt solution
