# Supplementary material for: Photochemical Restoration of Light Sensitivity in the Degenerated Canine Retina
Source: Pharmaceutics. 2022 Dec 3;14(12):2711. doi: 10.3390/pharmaceutics14122711 (PMC9783220; doi:10.3390/pharmaceutics14122711)
Supplement: Supplementary file 1 [file pharmaceutics-14-02711-s001.zip › Supplementary Table S3 List of dog retinas used for qRT-PCR.pdf]

Table S3: List of dog retinas used for qRT-PCR studies

Status	Dog ID-Eye	Sex	Age at collection (weeks)
WT	CEACMI-OS	F	24
WT	CEACID-OS	F	24
WT	CEACIV-OS	F	24
PDE6B mutant	2149-OS	F	22
PDE6B mutant	2150-OS	F	22
PDE6B mutant	2151-OS	F	22
RPGR mutant	Z478-OS	F	41
RPGR mutant	Z479-OS	F	41
RPGR mutant	Z480-OS	F	41

Abbreviations OD: right eye; OS: left eye; WT: wildtype; F: female
