# Supplementary material for: Photochemical Restoration of Light Sensitivity in the Degenerated Canine Retina
Source: Pharmaceutics. 2022 Dec 3;14(12):2711. doi: 10.3390/pharmaceutics14122711 (PMC9783220; doi:10.3390/pharmaceutics14122711)
Supplement: Supplementary file 1 [file pharmaceutics-14-02711-s001.zip › Supplementary Table S4 List of primers used for qRT-PCR.pdf]

Supplementary Table S4: List of primers used for qRT-PCR

Gene	Accession no.	Forward primer	Reverse primer
P2RX1	XM_005624967.2	CGTGGTTCTGGTCTACGTTATT	ACGGCTAGACCTTTGAGTTTC
P2RX2	XM_014108077.1	GTTCTCCAAGGGCAACATAGA	CCAGTTGATGACGACTCCAAT
P2RX3	XM_540614.3	TTCTTGCACGAGAAGGCTTAC	GTCCATGACTCTGTTGGCATAG
P2RX4	XM_003639907.3	TCAGGTTTGCCAAGTACTACAG	CAGCCTTTCCGAACACTATGA
P2RX5	XM_005624961.2	CAGACTTTCCTACCCACCTTAC	AGGCCACATGTGAAGTAGTG
P2RX6	XM_005636614.2	GGGATCTTCTGTATGCCTCTTC	TCCAGATGTACAGCCTCCTAT
P2RX7	XM_014107713.1	GAAGGTGAAGAAGTCCCAAGA	CTCCTCTGATTGTCCAGGAATAG
HCN1	XM_850162.3	TTCAACTGTCGGAAGCTAGTG	CCAGGTTGAAACACCTCAAATC
HCN2	XM_014121847.1	CCGTGGACTACATCTTCCTTATC	GGCTGAGGATCTTGGTGAAG
HCN3	XM_005622734.2	GGCAATACCAGGAGAAGTACAA	CCTGGTAGCGATGCTCATAAT
HCN4	XM_014109391.1	CACACCCTGGATTGTCTTCA	GGATGATCTCTGTGTTGTCCTC
GAPDH	NM_001003142.2	AACAGTGACACCCACTCTTC	CGGTTGCTGTAGCCAAATTC
