# Supplementary figures and images for: Photochemical Restoration of Light Sensitivity in the Degenerated Canine Retina
Source: Pharmaceutics. 2022 Dec 3;14(12):2711. doi: 10.3390/pharmaceutics14122711 (PMC9783220; doi:10.3390/pharmaceutics14122711)

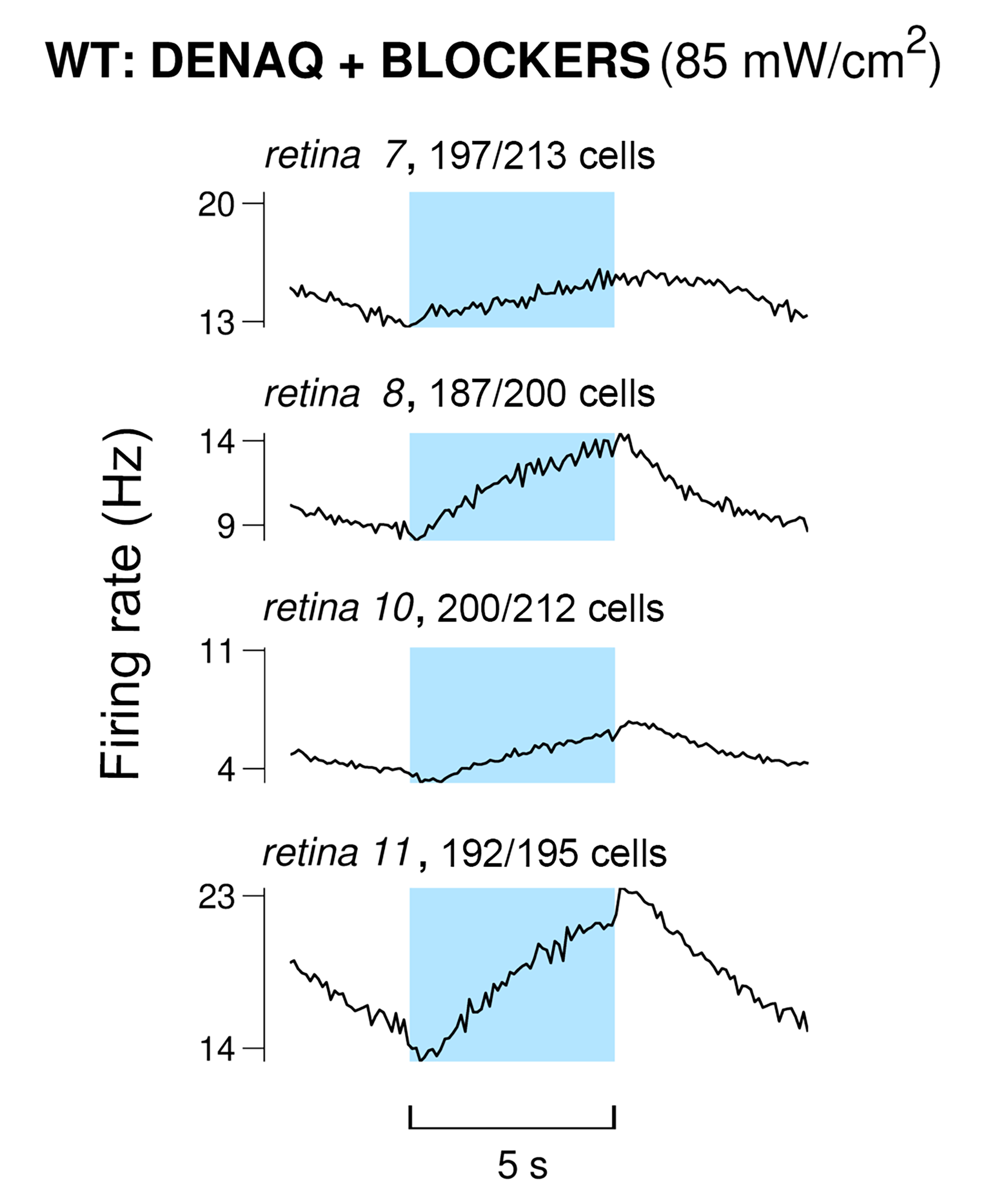

Supplement: Supplementary file 1 [file pharmaceutics-14-02711-s001.zip › Figure_S1.tif]

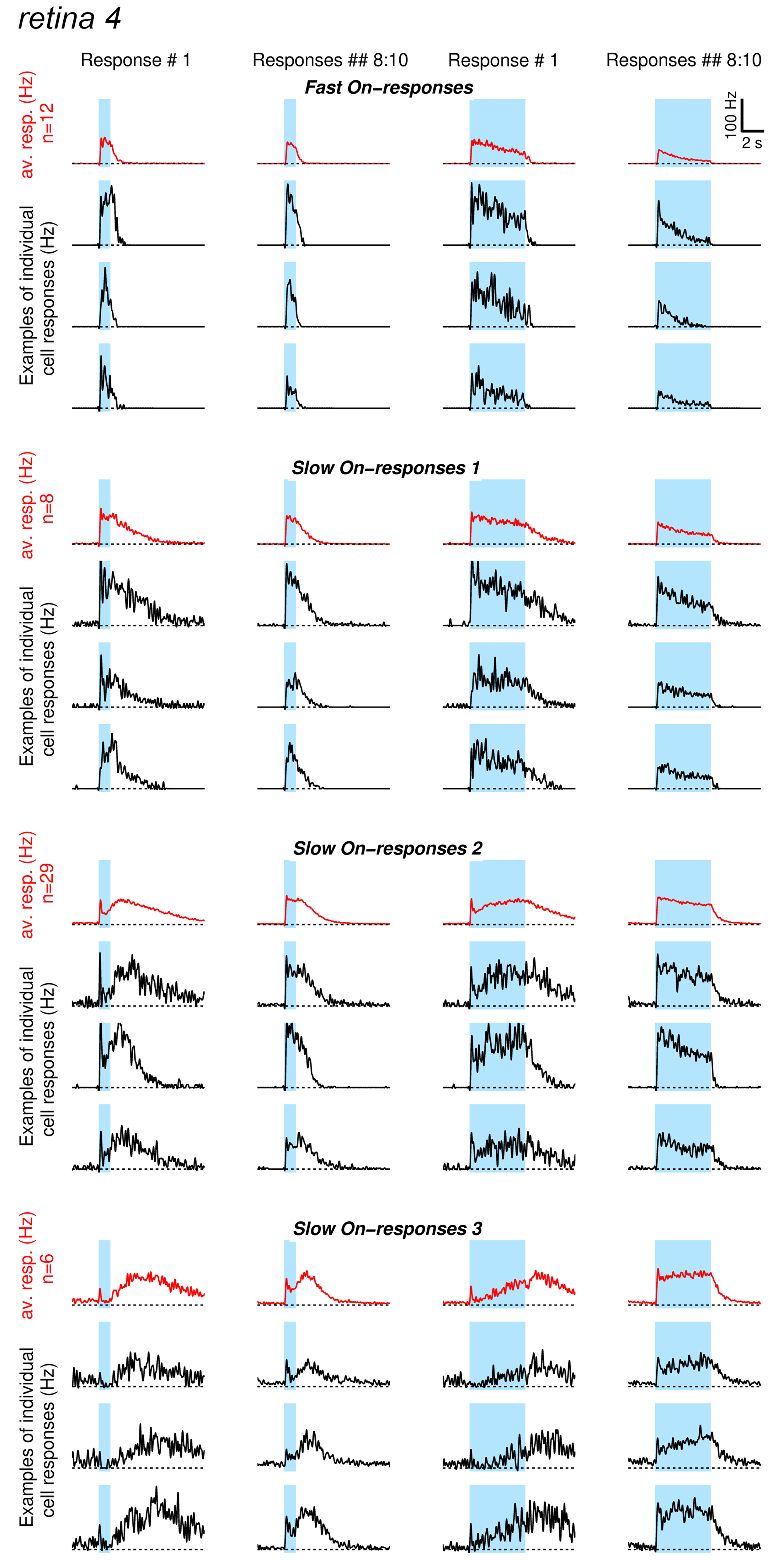

Supplement: Supplementary file 1 [file pharmaceutics-14-02711-s001.zip › Figure_S2.tif]

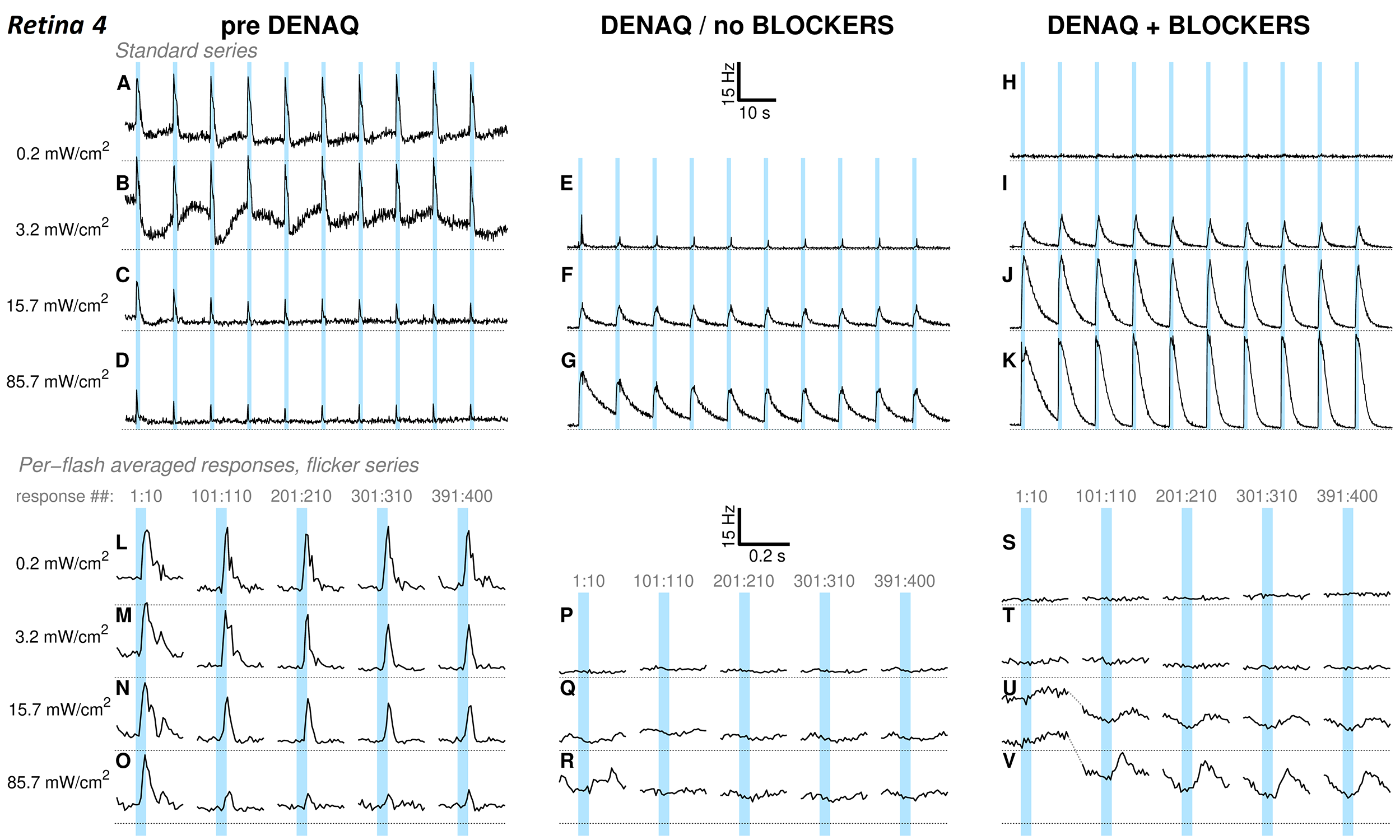

Supplement: Supplementary file 1 [file pharmaceutics-14-02711-s001.zip › Figure_S3.tif]

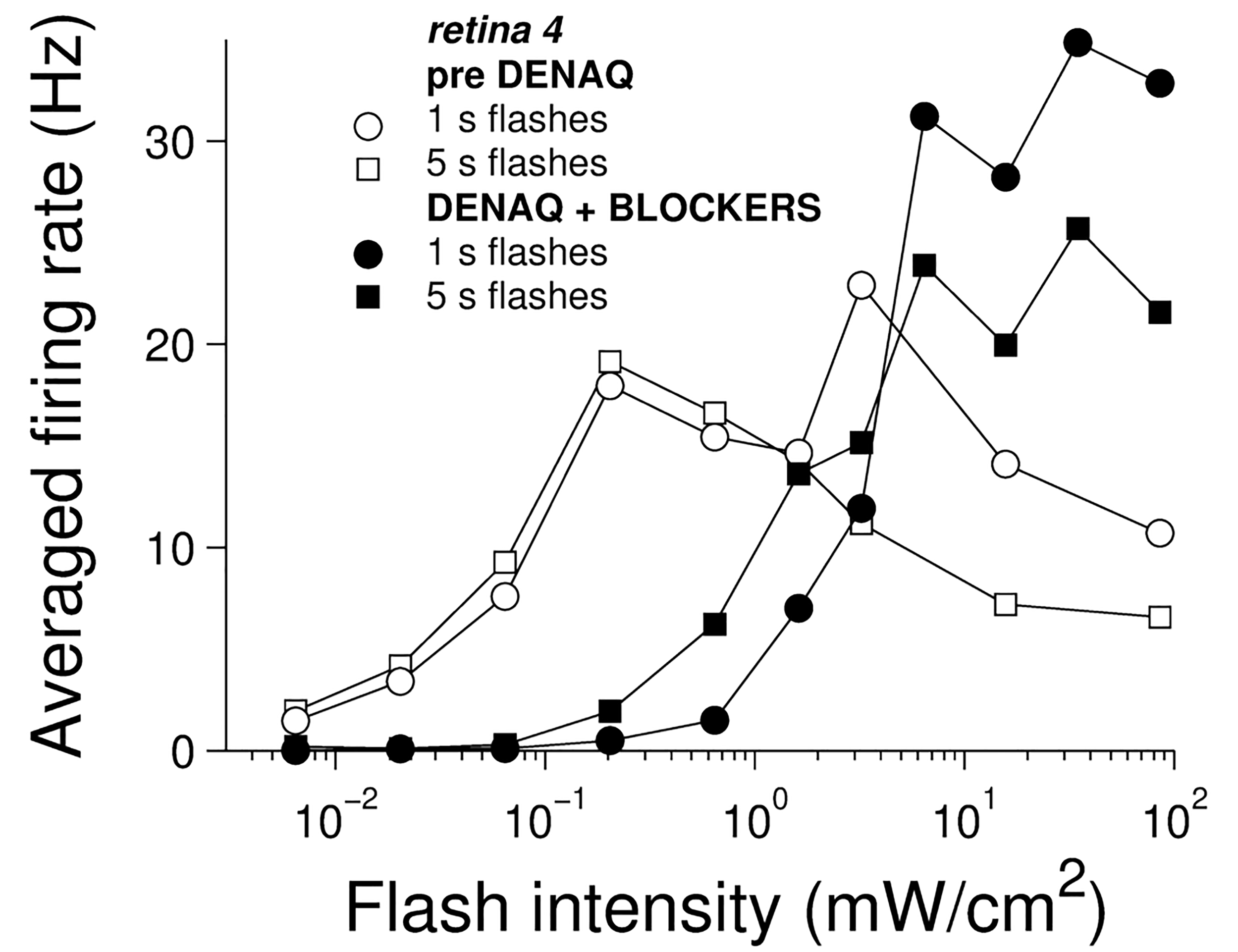

Supplement: Supplementary file 1 [file pharmaceutics-14-02711-s001.zip › Figure_S4.tif]

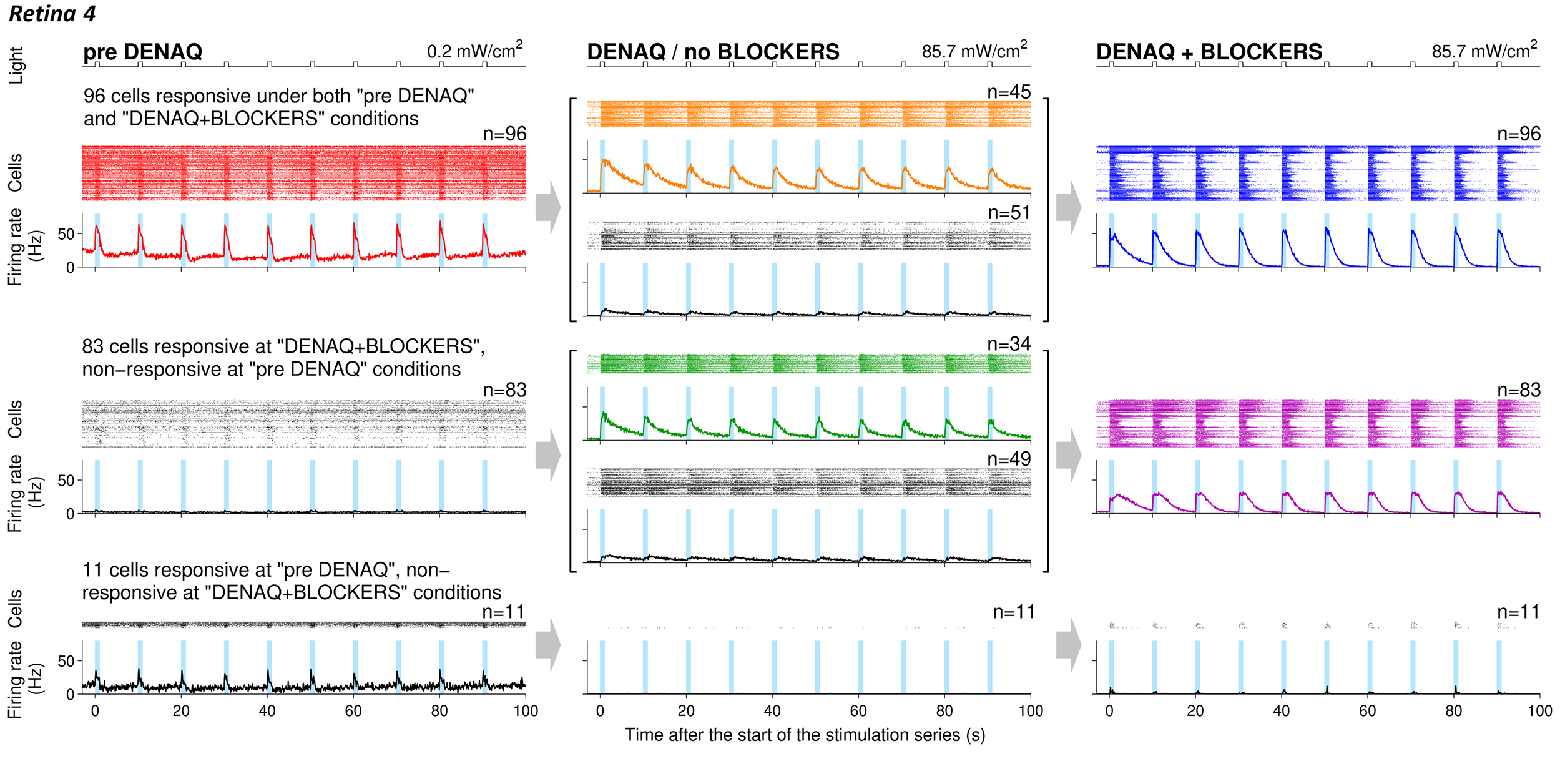

Supplement: Supplementary file 1 [file pharmaceutics-14-02711-s001.zip › Figure_S5.tif]
