# Supplementary material for: Photochemical Restoration of Light Sensitivity in the Degenerated Canine Retina
Source: Pharmaceutics. 2022 Dec 3;14(12):2711. doi: 10.3390/pharmaceutics14122711 (PMC9783220; doi:10.3390/pharmaceutics14122711)
Supplement: Supplementary file 1 [file Supplementary Table S1 List of retinas and conditions used for MEA.pdf › Supplementary Table S1 List of retinas and conditions used for MEA.pdf]

Table S1: List of retinas and experimental conditions used for MEA studies.

	Dog ID-Eye	Retina #	Status	Sex	Age (weeks)	Compound	Concentration (mM)	Volume (μL)	vehicle
**Ex vivo application:**									
	2205-OD	1	*PDE6B* mutant	M	31	DENAQ-ac.	1	1000	1% DMSO in Ringer's solution
	2073-OS	2	*PDE6B* mutant	F	111	DENAQ-ac.	1	1000	1% DMSO in Ringer's solution
	2073-OD	3	*PDE6B* mutant	F	111	DENAQ-Cl	1	1000	1% DMSO in Ringer's solution
	2065-OD	4	*PDE6B* mutant	F	282	DENAQ-ac.	1	1000	1% DMSO in Ringer's solution
	1911-OS	5	*PDE6B* mutant	F	403	DENAQ-ac.	1	1000	1% DMSO in Ringer's solution
	Z485-OS	6	*RPGR* mutant	F	56	DENAQ-ac.	1	1000	1% DMSO in Ringer's solution
	E1052-OS	7	WT	F	109	DENAQ-ac.	1	1000	1% DMSO in Ringer's solution
	E1052-OD	8	WT	F	109	DENAQ-ac.	1	1000	1% DMSO in Ringer's solution
	N289-OS	9	WT	F	54	DENAQ-ac.	1	1000	1% DMSO in Ringer's solution
	GI88-OS	10	WT	F	344	DENAQ-ac.	1	1000	1% DMSO in Ringer's solution
	GI88-OD	11	WT	F	344	DENAQ-ac.	1	1000	1% DMSO in Ringer's solution
	AS2-388-OS	12	WT	M	17	DENAQ-ac.	1	1000	1% DMSO in Ringer's solution
**In vivo (post vitrectomy) injection:**									
	2081-OS	13	*PDE6B* mutant	F	261	DENAQ-ac.	3	150	10% DMSO in BSS
	AS345-OD	14	*PDE6B* mutant	M	177	DENAQ-ac.	3	150	10% DMSO in BSS
	WM11-OS	15	*PDE6B* mutant	F	15	DENAQ-ac.	10	150	10% DMSO in BSS

Abbreviations: OD: right eye; OS: left eye; WT: wildtype; F: female; M: male; DENAQ-Cl: DENAQ-chloride; DENAQ-ac: DENAQ-acetate; DMSO: dimethyl sulfoxide; BSS: balanced salt solution
